# Cross-species and cross-platform gene expression studies with the Bioconductor-compliant R package 'annotationTools'

**DOI:** 10.1186/1471-2105-9-26

**Published:** 2008-01-17

**Authors:** Alexandre Kuhn, Ruth Luthi-Carter, Mauro Delorenzi

**Affiliations:** 1 Swiss Institute of Bioinformatics (SIB), CH-1015 Lausanne, Switzerland; 2 Brain Mind Institute, Ecole Polytechnique Fédérale de Lausanne, CH-1015 Lausanne, Switzerland; 3 National Center of Competence in Research (NCCR) Molecular Oncology, Swiss Institute of Experimental Cancer Research (ISREC), CH-1066 Epalinges, Switzerland

## Abstract

**Background:**

The variety of DNA microarray formats and datasets presently available offers an unprecedented opportunity to perform insightful comparisons of heterogeneous data. Cross-species studies, in particular, have the power of identifying conserved, functionally important molecular processes. Validation of discoveries can now often be performed in readily available public data which frequently requires cross-platform studies.

Cross-platform and cross-species analyses require matching probes on different microarray formats. This can be achieved using the information in microarray annotations and additional molecular biology databases, such as orthology databases. Although annotations and other biological information are stored using modern database models (e.g. relational), they are very often distributed and shared as tables in text files, i.e. flat file databases. This common flat database format thus provides a simple and robust solution to flexibly integrate various sources of information and a basis for the combined analysis of heterogeneous gene expression profiles.

**Results:**

We provide annotationTools, a Bioconductor-compliant R package to annotate microarray experiments and integrate heterogeneous gene expression profiles using annotation and other molecular biology information available as flat file databases. First, annotationTools contains a specialized set of functions for mining this widely used database format in a systematic manner. It thus offers a straightforward solution for annotating microarray experiments. Second, building on these basic functions and relying on the combination of information from several databases, it provides tools to easily perform cross-species analyses of gene expression data.

Here, we present two example applications of annotationTools that are of direct relevance for the analysis of heterogeneous gene expression profiles, namely a cross-platform mapping of probes and a cross-species mapping of orthologous probes using different orthology databases. We also show how to perform an explorative comparison of disease-related transcriptional changes in human patients and in a genetic mouse model.

**Conclusion:**

The R package annotationTools provides a simple solution to handle microarray annotation and orthology tables, as well as other flat molecular biology databases. Thereby, it allows easy integration and analysis of heterogeneous microarray experiments across different technological platforms or species.

## Background

DNA microarrays allows reliable measures of the levels of several tens of thousands of different mRNAs simultaneously [1]. The microarray 'annotation' contains information specifying the mRNAs and genes assayed by the probes present on a particular microarray. A good annotation not only lists the probed transcripts but also contains biological information about the corresponding genes and information about the reliability of each probe-transcript association for instance. The information needed to define an annotation is extracted from molecular biology databases, such as Entrez Gene [2].

In the case of commercial microarrays supported by a dedicated analysis software (e.g. GCOS for Affymetrix arrays [3]), an annotation is provided by the manufacturer and integrated in the analysis environment. On the other hand, if custom analyses are performed with the help of a versatile computing environment like R [4], an annotation for the microarray format of interest can be chosen explicitly. Here, we present an R package to support the use of annotations and other molecular biology databases provided as flat file databases (i.e. tables in plain text files). Flat annotation databases may be provided by microarray manufacturers or generated anew on the basis of microarray probe sequences by researchers who aim at a better definition of targeted transcripts (in particular for short oligonucleotide arrays, see e.g. [5]).

Some functions in annotationTools are tailored to mine flat file databases containing gene homology/orthology information. DNA microarrays are available for numerous different species allowing for the comparison of gene expression levels across different organisms. This opportunity is promising [6-8], in particular for the better understanding of human diseases, where the comparison of disease-related expression profiles in humans and in animal models might yield important insights into pathological molecular mechanisms (see e.g. [9]). Furthermore, comparing a disease condition in humans and in animal models, we can assess whether experimental models recapitulate aspects of the human disease [10], a critical step in validating disease models with regard to their use in preclinical therapeutical trials for instance. The comparison of transcriptional profiles across different species can make use of orthologous genes (i.e. two genes that derive from a single gene in the last common ancestor of the two species), assuming that they have retained the same function and are thus involved in similar processes in the two species. Whole-genome sequences have allowed extensive mapping of orthologs across many species, and several databases store clusters of homologous/orthologous genes (e.g. HomoloGene [11]).

Annotations and orthology information are sufficient to perform cross-species analyses but combining heterogeneous sources of information is often arduous (e.g. [12]). Based on a set of functions with common input and output formats and mining flat databases, annotationTools implements a robust solution for integrating heterogeneous data. To illustrate the use and functionality of our software, we will present the following example analyses that are directly relevant to cross-species mRNA profiling studies. We mapped probes across two widely used microarray platforms (Affymetrix and Illumina) and also mapped probes on a mouse microarray to their orthologs on a human microarray using information supplied by HomoloGene. We further show how the latter mapping differed from mappings obtained using 3 alternative sources of orthology information (i.e. Affymetrix, Ensembl [13] or EGO [14]). Building on this example and using data from human patients and from a murine disease model, we then demonstrate how annotationTools can be used to easily perform cross-species comparisons of gene expression profiles.

## Implementation

Functions in annotationTools are coded in pure R and do not depend on other R packages. They are therefore easily usable on any R installation. The annotation functions accept a vector of identifiers to be annotated (e.g. probe identifiers) and an annotation table, created by loading a flat annotation file in a data.frame object (see also Results). In the annotation table, each column contains a piece of annotation information (like e.g. probe identifier, gene symbol or chromosomal location) and each row contains a probe record. The identifiers to be annotated are looked up in the annotation table. Since a single identifier can be annotated with multiple items (like a probe hybridizing to several transcripts and therefore annotated with several different gene symbols), the annotation retrieved for the whole vector of identifiers is output as a list where the i-th element is a vector containing all annotation items found for the i-th input identifier. Options can be set to output the cause of an annotation failure: empty or invalid input identifier, input identifier not found in the annotation table, input identifier with no annotation information provided in the annotation table.

The annotation functions can be grouped according to their look-up behavior. First, functions that find a single match in the annotation table for each input identifier (i.e. the first occurrence of the identifier in the table): getANNOTATION, getGENEID, getGENESYMBOL, getGENETITLE, getGO. Note that getANNOTATION is a flexible function that can be set to look up any fields (column) in an arbitrary annotation table. The four other functions are tailored to accept annotation tables provided by Affymetrix but they can be easily customized to other formats by setting options.

A second group of annotation functions find multiple matches (i.e. multiple records) for each input identifier: getMULTIANNOTATION, getPROBESET and getHOMOLOG. getMULTIANNOTATION is a flexible annotation function that can work with arbitrary annotation tables whereas getPROBESET works with Affymetrix annotation tables by default. getHOMOLOG is designed to look up homologs or orthologs and accepts an homology/orthology table and two inputs: a vector of gene identifiers and a target species identifier.

Finally, a third group of functions making use of the previous two groups allows to perform higher-level operations (like e.g. generating mapping tables of orthologous probes). In conclusion, our package provides the user with a set of customizable functions with consistent input and output formats that can be easily chained to perform more complex operations.

## Results

### Examples of basic annotation operations

Let us consider a typical Affymetrix GeneChip experiment and assume that we chose to use the annotation provided by Affymetrix. After downloading the annotation from Affymetrix website (e.g. for array format HG-U133 Plus 2.0, the file 'HG-U133_Plus_2_.naXX.annot.csv', where 'XX' indicates the version number, see [15]), the user loads it into R as a data.frame with

> annotation <- read.csv("HG-U133_Plus_2.naXX.annot.csv", colClasses="character")

Genes associated with particular probe sets can then be retrieved with the function getGENESYMBOL. To retrieve genes whose transcripts are probed by probe sets '117_at' and '1007_s_at', type

> getGENESYMBOL(c('117_at','1007_s_at'), annotation)

where the variable 'annotation' is the data.frame containing Affymetrix annotation. Being interested in human gene RFC2 (Entrez geneID 5982), we can find its mouse ortholog using the information in HomoloGene. Having downloaded the flat database [11] to a file called 'homologene.data' for instance, we first load it into R and then mine it using the function getHOMOLOG

> homologene <- read.delim('homologene.data', header = FALSE)

> getHOMOLOG(5982, 10090, homologene)

where the variable 'homologene' is the data.frame containing the HomoloGene database and 10090 is the taxonomy ID of *Mus musculus *[16]. Detailed function descriptions and further examples are given in help files and in the package's vignette.

### Cross-platform probe mapping

**Figure 1:**
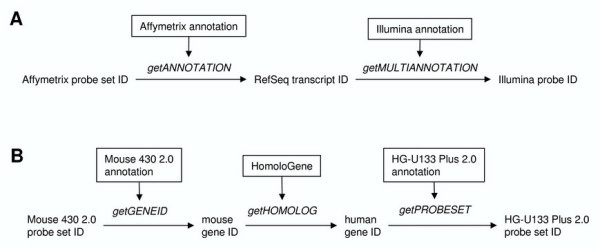
Strategies to perform an Affymetrix to Illumina cross-platform mapping and an Affymetrix to Affymetrix cross-species mapping (A and B respectively, see text for details). Probe, RefSeq and gene identifiers (upright characters) are mapped using various functions in annotationTools (italic characters) that mine different databases (boxed text).

As an illustration of the use and application of functions in annotationTools, we next outline how to perform a cross-platform mapping of probes. Suppose that we would like to compare the outcome of two gene expression profiling experiments, one performed with the Affymetrix Mouse 430 2.0 array and the other with the Illumina Mouse-6 array. We thus need to find pairs of probes measuring the same transcript on both platforms. Affymetrix and Illumina provide annotation information including the transcript measured by each probe, designated by its RefSeq accession number [17]. This identifier can thus be used to find matching probes across platforms, as illustrated in Figure 1A: we first use the multi-purpose function getANNOTATION on Affymetrix annotation to retrieve RefSeq accession numbers corresponding to Affymetrix probe sets and second, use those to retrieve Illumina probes (also called targets) annotated with the same corresponding RefSeq accession numbers, mining Illumina annotation with the help of getMULTIANNOTATION. Since several probes can measure the same transcript and are thus annotated with the same RefSeq accession number, in this case this function is to be preferred over getANNOTATION that will return the first match only (see Implementation).

Using this strategy to map all 45'101 probe sets present on Affymetrix Mouse 430 2.0 to Illumina Mouse-6v10, we found 31'830 (71%) probe sets with at least one matching probe on Illumina array (corresponding annotation file was obtained from Illumina, see [18]). The first 10 probe sets in Affymetrix annotation and their matching Illumina probes are shown in Table 1. Gene symbols (derived from Affymetrix and Illumina annotations, column 3 and 6 respectively) are for illustration purpose and were not used for finding probe pairs. The first Affymetrix probe set (1415670_at) was annotated with two RefSeq identifiers but only one of those (NM_017477) was found to match an Illumina probe. Conversely, the second and ninth Affymetrix probe sets, each annotated to a single RefSeq identifier, matched to three, respectively two different Illumina probes annotated with the same RefSeq identifier.

**Table 1:** Mapping of the first 10 probe sets on Affymetrix Mouse 430 2.0 to probes (also called targets) annotated with the same RefSeq IDs on Illumina Mouse-6v10. Gene symbols (GS) for Affymetrix and Illumina (column 3 and 6 respectively) are indicative and were not used to perform the mapping. In this example, we disregarded the version number of RefSeq identifiers (i.e. the suffix of RefSeq accession number) and considered accession number with the same prefix (i.e. NM_123456.1 and NM_123456.2 for instance) to be equivalent.

**Affy probe set**	**RefSeq (Affy)**	**GS (Afffy)**	**Illumina target**	**RefSeq (Illumina)**	**GS (Illumina)**
1415670_at	NM_017477, NM_201244	Copg	scl29780.26_164-S	NM_017477	Copg
1415671_at	NM_013477	Atp6v0d1	scl011972.1_247-S, scl000737.1_18-S, scl000708.1_100-S	NM_013477	Atp6v0d1
1415672_at	NM_020585	Golga7	scl057437.1_41-S	NM_020585	Golga7
1415673_at	NM_133900	Psph	scl25996.9.1_198-S	NM_133900	Psph
1415674_a_at	NM_021789	Trappc4	scl35956.2.1_28-S	NM_021789	Trappc4
1415675_at	NM_010073	Dpm2	scl013481.4_295-S	NM_010073	Dpm2
1415676_a_at	NM_011186	Psmb5	scl019173.2_9-S	NM_011186	Psmb5
1415677_at	NM_026819	Dhrs1	scl45528.8.1_39-S	NM_026819	Dhrs1
1415678_at	NM_008910	Ppm1a	scl0019042.1_40-S, scl019042.1_95-S	NM_008910	Ppm1a
1415679_at	NM_025498	Psenen	scl066340.3_30-S	NM_025498	1700023M09Rik

The use of gene symbols was not always consistent across both annotations, as illustrated by the last probe set pair annotated to *Psenen *(official symbol) by Affymetrix and to *1700023M09Rik *(synonym) by Illumina. This suggests that, in this example, matching probes via gene symbols instead of RefSeq identifiers would have yielded an incomplete mapping, which highlights the importance of the matching identifier when setting up a mapping strategy. Note that if annotations do not contain any suitable identifier to be used as matcher, an intermediate mapping step can be introduced that makes use of a molecular biology database providing a link between several identifiers (e.g. Entrez Gene, Unigene). In the mapping presented above, imagine for instance that one of two microarray annotations would have listed gene IDs and not RefSeq IDs, we could have used the database gene2accession (provided by Entrez Gene via FTP [19]) to link these two identifier types through an additional, intermediate matching step.

### Mapping of orthologous Affymetrix probe sets

To illustrate how annotationTools allows to combine various sources of information, we now consider the mapping of orthologous probe sets (i.e. probe sets probing transcripts from orthologous genes) across two Affymetrix GeneChip formats, namely from Mouse 430 2.0 to HG-U133 Plus 2.0. We used the following procedure, illustrated in Figure 1B: annotate probe sets on Mouse 430 2.0 with their gene IDs using the corresponding Affymetrix annotation and use the gene IDs to mine HomoloGene and find orthologous gene IDs in the target species (Homo sapiens). Finally, use the Affymetrix annotation for HG-U133 Plus 2.0 to retrieve the corresponding (orthologous) probe sets. This method is easily implemented using getGENEID, getHOMOLOG and getPROBESET sequentially (see Figure 1B and the example code in the package's vignette) or, alternatively through a single call to the wrapper function ps2ps

> ps2ps(annotation_HGU133Plus2, annotation_Mouse4302, homologene, 10090)

assuming that 'annotation_HGU133Plus2', 'annotation_Mouse4302' and 'homologene' are data.frame objects containing the corresponding annotations and the HomoloGene database. 10090 is the taxonomy ID of *Mus musculus*. The function ps2ps returns a complete mapping table as a data.frame. This operation lasts on the order of ten minutes on a desktop computer. We compared the result of this procedure to 3 alternative sources of orthologous Affymetrix probe sets: Resourcerer (an interface to the Eukaryotic Gene Ortholog database), Ensembl, and Affymetrix orthology file ('Mouse430_2_ortholog.csv'). The 3 corresponding mapping tables (obtained as text files) can be easily mined for orthologous probe sets with getHOMOLOG.

**Figure 2:**
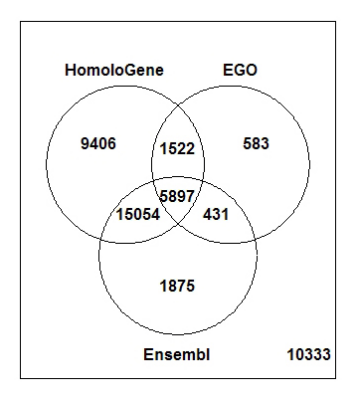
Venn diagram showing the number of Affymetrix Mouse 430 2.0 probe sets mapped to at least one probe set on Affymetrix HG-U133 Plus 2.0 using the HomoloGene-based method, EGO or Ensembl.

**Figure 3:**
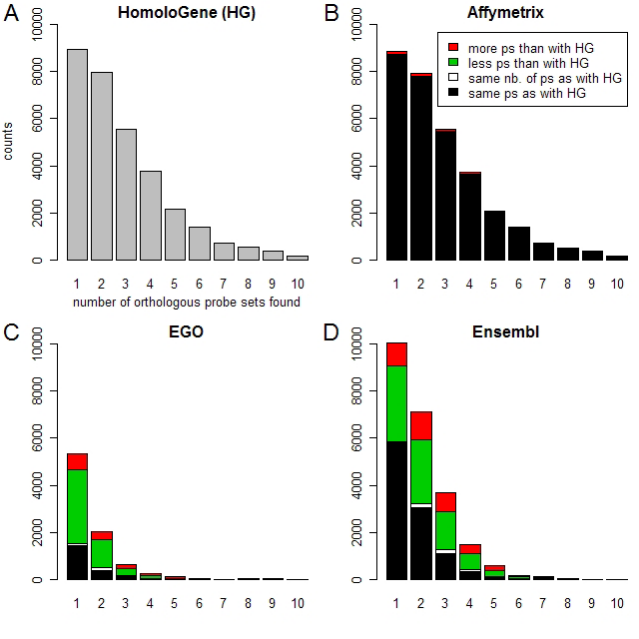
Number of orthologous probe sets found on Affymetrix array HG-U133 Plus 2.0 for each probe set on Affymetrix array Mouse 430 2.0, using various orthology databases. (A) We mapped all probe sets on array Mouse 430 2.0 (annotation file version 12/19/2005) to their orthologs on HG-U133 Plus 2.0 (annotation file version 11/10/2004), using HomoloGene (version 08/11/2006) and compared with the corresponding orthologous probe sets given by (B) Affymetrix ortholog file 'Mouse430_2_ortholog.csv' (version 07/12/2006) (C) EGO (release 9.0) and (D) Ensembl (as of 08/15/2006). In panels B-D, stacked color bars in each class indicate, for each mouse probe set, if more (red), less (green), the same number (white), or the exact same orthologous probe sets (black) were found as with HomoloGene. The distributions in panels A-D are displayed between 1 and 10. Note that all database versions used in this example are not current versions anymore, EGO in particular having undergone a major rebuild.

31'879 mouse probe sets (from a total of 45'101 on Mouse 430 2.0 array) were mapped to 1 or more orthologous human probe sets on HG-U133 Plus 2.0 array using our HomoloGene-based procedure, 31'589 were mapped using Affymetrix ortholog file, 23'257 using Ensembl and 8'433 using EGO. Figure 2 shows the number of mouse probe sets mapped to at least one human probe set by the different methods (excluding Affymetrix for its similarity to the HomoloGene-based method, see below). 9406 mouse probe sets could be mapped to at least 1 ortholog using HomoloGene only (i.e. neither Ensembl nor EGO provided any ortholog probe sets for these). The opposite was true for 1875 and 583 mouse probe sets, for which Ensembl and EGO respectively, but no other methods, found at least one orthologous probe set. Overall, 10'333 mouse probe sets could not be mapped using any of the three methods. Figure 3A displays the distribution of the number of orthologous human probe sets found (between 1 and 10) for each mouse probe set using our HomologGene-based procedure. For comparison, Figure 3B–D represent the same distribution but using Affymetrix ortholog file, EGO and Ensembl, respectively. Affymetrix ortholog file returned the exact same set of human orthologous probe sets as the HomoloGene-based method for most of the mouse probe sets (97%, colored black on Figure 3B). Affymetrix actually uses the information in HomoloGene to build its orthology files (Salomone J-Y, Affymetrix, personal communication), such that the small differences between both methods can be assumed to be due to the use of different HomoloGene versions. Furthermore, using Ensembl and EGO, and for mouse probe sets with at least one ortholog in human, on average less orthologs were found than with the HomoloGene-based method (as shown by the longer green and shorter red bars in every stacks in Figure 3C–D).

### Cross-species analysis of gene expression changes

We now present a practical example of cross-species analysis using gene expression data from Huntington's disease patients and from a genetic mouse model of the disease. Huntington's disease (HD) is a neurological disorder caused by a trinucleotide (CAG) expansion in the *HD *gene. Animal models of HD have allowed the demonstration that mutant protein expression results in transcriptional dysregulation of many genes [20]. More recently, many mRNA changes have also been detected in the brains of HD patients [21]. How do these changes compare with those identified in mouse models?

Here we will consider the CHL2 mouse model of HD [22] and investigate whether the most robust mRNA changes detected in striatal samples of these mutant mice parallel those measured in the corresponding brain region of HD patients. Thereby, we aim at assessing the faithfulness of the animal model with regard to transcriptional dysregulations in human. We used public data from a study that profiled striata of CHL2 and control mice and assessed differential expression between both genotypes [23]. In short, Strand et al. extracted RNA from 3 transgenic and 3 control animals and hybridized it to Affymetrix MG-U74Av2 arrays. These were normalized using RMA [24] and differential expression analysis was performed with the R package limma [25]. The list of Affymetrix probe sets along with measures of expression change and associated statistics for differential expression is publicly available [26]. In addition, we used data from a large transcriptomic study of human HD using Affymetrix HG-U133A arrays [21]. Human striatal gene expression profiles from 44 HD patients and 36 controls were analyzed similarly as described for the mice and the list of probe sets with associated measures of expression change and differential expression statistics is available from the same source [26].

To perform this mouse-human comparison, we first needed to find orthologous probe sets on the two microarrays used in the aforementioned studies (namely MG-U74Av2 for the mouse and HG-U133A for humans). As presented above, a table of orthologous probe sets can easily be generated with the function ps2ps in annotationTools. This mapping table and results from differential gene expression analyses of the mouse and human data could then be used to look up the probe sets present in the human data that are orthologous to the selected top mouse probe sets (i.e. those detecting differential gene expression in the CHL2 model). This is implemented by the function getOrthologousProbesets

> getOrthologousProbesets(mouse_ps, human_diffExpr, mapping)

that takes a vector of probes to be matched ('mouse_ps'), a data.frame object containing the target probe sets and associated log_2 _fold changes or statistics of interest ('human_diffExpr'), and the mapping table ('mapping'). This returns a list of target probe sets and associated values (log_2 _fold changes, respectively statistics). In case of multiple human orthologous probe sets found to match a given mouse probe set, the function can be configured to either output a summary value (e.g. median log_2 _fold change) or a single value corresponding to a selected probe set (e.g. the value associated with the most significant probe set, see the corresponding help file for a complete description of the function and its options). This information allowed us to readily compare gene expression changes in the CHL2 mouse model with those measured in HD patients.

**Figure 4:**
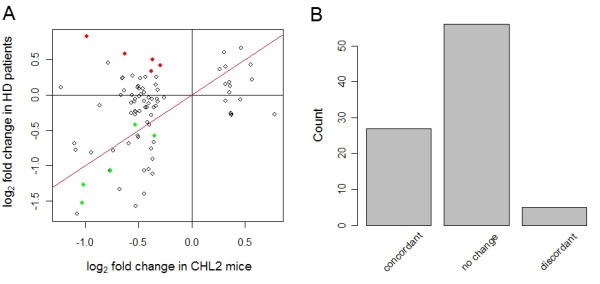
A. Gene expression regulations (log_2 _fold changes) measured by the top 100 mouse probe sets in the CHL2 mouse model of Huntington's disease and their orthologous regulations measured in the brain of human HD patients. The red line is the plot diagonal. In case multiple human probe sets were found for a given mouse probe set, we selected the probe set with the smallest p-value (i.e. detecting the most robust change in HD patients versus controls). Note that the probe set selection method can be specified by the user (see package's vignette). Green and red dots indicate the 5 most significant changes in CHL2 mice showing concordant, respectively discordant regulations in HD patients. B. 88 ortholog pairs (out of the 100 mouse probe sets showing the most significant changes) were classified according to the concordance of HD-related differential expression measures in mice and humans. 'No change' indicate ortholog pairs for which the human gene did not show significant differential expression between HD patients and controls.

Figure 4A presents the result of this analysis and shows the log_2 _fold change in expression for the top 100 gene expression changes in CHL2 mice against orthologous regulations measured in HD patients. In case of multiple orthologous human probe sets, we selected the probe set measuring the most significant expression change. 88 human orthologous probe sets were found in the human data (out of 100 mouse probe sets) and the overall correlation measured by Kendall's tau was 0.2. To summarize this comparison, we considered the direction of regulation detected in patients and disregarded its magnitude. Thereby we could sort each of the 88 mouse-human probe set pairs to one of three categories: first, 'concordant' pairs were defined as showing a significant expression change in human (i.e. Bonferroni corrected p-value < 0.05) and in the same direction as for the mouse model. Second, 'discordant' pairs described those with a significant expression change in human (i.e. Bonferroni corrected p-value < 0.05) but in the opposite direction compared to mice. Third, 'no change' pairs in which the human orthologous gene did not show a significant expression change (i.e. Bonferroni corrected p-value >= 0.05). A large fraction of 'concordant' pairs thus indicates recapitulation of the human HD signature by the murine model. We observed about five times more 'concordant' than 'discordant' pairs among the 88 mouse-human orthologs (Figure 4B). The majority (56) of ortholog pairs, however, did not detect a significant expression change in humans, although they were ranked among the 100 most significant expression changes in mouse. The five most significant changes in mouse showing 'concordant' or 'discordant' regulations in humans are listed in Table 2 and displayed as green, respectively red dots in Figure 4A. Note that these genes did not consistently display the largest (absolute) change in expression, reflecting variable expression within experimental groups in the mouse data.

**Table 2:** Probe sets associated with the 5 most significant mRNA changes in mice and whose human ortholog showed significant same-sign regulation (i.e. concordant, A) or significant different-sign regulation (i.e. discordant, B). PS, GS, logFC and P stand for probe set, gene symbol, log_2 _fold change and p-value respectively. P-values in the human dataset (last column) are Bonferroni-corrected to adjust for multiple testing (44'928 human probe sets in total).

**A: concordant**							
**mouse PS**	**mouse GS**	**mouse logFC**	**mouse P**	**human PS**	**human GS**	**human logFC**	**human corrected P**

93273_at	Snca	-0.53	6.8E-04	204467_s_at	SNCA	-0.42	6.8E-05
96497_s_at	Myt1l	-1.03	8.0E-04	210016_at	MYT1L	-1.53	1.8E-04
99511_at	Prkcb	-1.02	8.6E-04	209685_s_at	PRKCB1	-1.27	2.7E-05
102711_at	Rgs14	-0.76	1.0E-03	211021_s_at	RGS14	-1.07	1.7E-08
104678_at	Gas7	-0.35	1.9E-03	202192_s_at	GAS7	-0.58	8.1E-05

**B: discordant**							

**mouse PS**	**mouse GS**	**mouse logFC**	**mouse P**	**human PS**	**human GS**	**human logFC**	**human corrected P**

102704_at	Aqp4	-0.99	3.3E-03	210068_s_at	AQP4	0.84	4.9E-02
102299_at	Prkca	-0.38	6.8E-03	213093_at	PRKCA	0.34	3.2E-02
100003_at	Ryr1	-0.63	6.8E-03	205485_at	RYR1	0.59	9.6E-04
100888_at	Sorl1	-0.37	9.5E-03	203509_at	SORL1	0.50	3.0E-04
99367_at	5530600P05Rik	-0.29	1.2E-02	200713_s_at	MAPRE1	0.43	1.6E-03

## Discussion

As shown in a recent study, different microarray technologies can reliably and concordantly measure gene expression profiles [1]. The combination of heterogeneous microarray datasets can subserve different purposes. On the one hand, one might want to combine several independent profiling experiments aimed at answering the same question but performed with different technologies. We have shown here how to use annotationTools to quickly map probes across different microarray platforms and thus 'align' datasets. Such parallel experiments can then be considered in the framework of meta-analysis and analyzed so as to reach robust conclusions that would have been difficult to obtain based on any single study (see e.g. [27,28]). Note that different statistical methods have been proposed for combining multiple independent studies, based on the use of parameter estimates (e.g. differential expression), effect sizes (e.g. standardized differential expression), p-values, statistic ranks or test decisions (e.g. via Venn diagrams). Their extension and application in the field of microarray studies is an area of current active research.

Alternatively, heterogeneous datasets can be considered sequentially: a first study is considered as a reference and is used to derive a particular transcriptional signature (e.g. for a given cellular pathway, cell population or disease) whose presence is then tested for in a second dataset. In this context, we showed how to use annotationTools to map orthologous probes across species and explore the recapitulation of a human disease signature in a particular mouse model. Our example suggested that such comparisons can be made at various resolution levels (e.g. correlation of differential expression measures, concordance of regulation direction, see Figure 4). Our package does not address the issue of significance testing in such comparisons. Over the last years, several groups have proposed solutions to this issue (e.g [29,30]) and a general framework for statistical testing of global similarity is now emerging [31,32]. We have recently proposed the concordance coefficient as a new measure of similarity between datasets (based on the concordance of gene expression, see Results and Figure 4B), that is amenable to formal statistical testing and that we used to assess the extent to which transcriptomic changes in Huntington's disease were recapitulated by different genetic mouse models [10]. In particular, we could show that the CHL2 model significantly recapitulated aspects of gene expression changes detected in HD patients.

An alternative Bioconductor [33] solution for annotation and ortholog finding makes use of specialized data packages, which are compilations of biological information obtained from various databases for probe sets of particular microarray formats. These meta-data packages are prepared with the package annBuilder and can be obtained from the Bioconductor website [34]. They can be subsequently queried with special functions available through the package annotate. Another Bioconductor solution is provided by the package biomaRt which allows the (online) query of biomart databases (like e.g. Ensembl). Annotation and homology/orthology information can thus be retrieved from the newest built of available databases without downloading the database. Many annotations and molecular biology databases, however, are readily (and sometimes exclusively) available as flat file databases. These include annotations provided by commercial microarray manufacturers, by academic facilities producing and annotating their own spotted arrays, as well as re-annotation efforts aimed at providing more faithful representation of the transcript species measured by a given microarray platform (e.g. [5]). Moreover, most information present in very large databases such as Entrez Gene for instance is distributed as various flat file databases. The package annotationTools provides the R user with a simple solution to mine and combine data from flat file databases in a systematic way. In particular, the functions handling orthology databases allow for a straightforward use of publicly available orthology information (e.g. HomoloGene). As cross-species studies will become more frequent in the near future, it is of particular importance to develop user-friendly, flexible analysis tools that ease the comparison of gene expression profiles across microarray platforms.

## Conclusion

The Bioconductor-compliant package annotationTools allows analysts to perform microarray annotation tasks, match orthologous probes across microarrays and, more generally, use and combine information from flat databases within R. In particular, it offers an easy solution for implementing cross-species analysis of gene expression, which is of timely relevance.

## Availability & requirements

The R package annotationTools is freely available under the GPL license and can be downloaded from Bioconductor. The details for this package are provided below.

Project name: annotationTools

Project home page: 

Operating systems: Linux, Windows

Programming language: R

Other requirements: none

License: GNU GPL

Restriction to use by non-academics: none

## Authors' contributions

AK conceived the package, wrote the code, performed data analysis and wrote the manuscript. RLC provided microarray data, input on data analysis and comments on the manuscript. MD contributed to the package design and data analysis and participated in the writing of the manuscript. All authors read and approved the final manuscript.
